# Detection and categorization of bacteria habitats using shallow linguistic analysis

**DOI:** 10.1186/1471-2105-16-S10-S5

**Published:** 2015-07-13

**Authors:** İlknur Karadeniz, Arzucan Özgür

**Affiliations:** 1 Department of Computer Engineering, Boğaziçi University, İstanbul, Turkey

**Keywords:** BioNLP Shared Task, bacteria biotopes, bacteria habitats, shallow syntactic analysis, ontology-based annotation, relation extraction, anaphora resolution, information extraction, text mining, Natural Language Processing

## Abstract

**Background:**

Information regarding bacteria biotopes is important for several research areas including health sciences, microbiology, and food processing and preservation. One of the challenges for scientists in these domains is the huge amount of information buried in the text of electronic resources. Developing methods to automatically extract bacteria habitat relations from the text of these electronic resources is crucial for facilitating research in these areas.

**Methods:**

We introduce a linguistically motivated rule-based approach for recognizing and normalizing names of bacteria habitats in biomedical text by using an ontology. Our approach is based on the shallow syntactic analysis of the text that include sentence segmentation, part-of-speech (POS) tagging, partial parsing, and lemmatization. In addition, we propose two methods for identifying bacteria habitat localization relations. The underlying assumption for the first method is that discourse changes with a new paragraph. Therefore, it operates on a paragraph-basis. The second method performs a more fine-grained analysis of the text and operates on a sentence-basis. We also develop a novel anaphora resolution method for bacteria coreferences and incorporate it with the sentence-based relation extraction approach.

**Results:**

We participated in the Bacteria Biotope (BB) Task of the BioNLP Shared Task 2013. Our system (Boun) achieved the second best performance with 68% Slot Error Rate (SER) in Sub-task 1 (Entity Detection and Categorization), and ranked third with an F-score of 27% in Sub-task 2 (Localization Event Extraction). This paper reports the system that is implemented for the shared task, including the novel methods developed and the improvements obtained after the official evaluation. The extensions include the expansion of the OntoBiotope ontology using the training set for Sub-task 1, and the novel sentence-based relation extraction method incorporated with anaphora resolution for Sub-task 2. These extensions resulted in promising results for Sub-task 1 with a SER of 68%, and state-of-the-art performance for Sub-task 2 with an F-score of 53%.

**Conclusions:**

Our results show that a linguistically-oriented approach based on the shallow syntactic analysis of the text is as effective as machine learning approaches for the detection and ontology-based normalization of habitat entities. Furthermore, the newly developed sentence-based relation extraction system with the anaphora resolution module significantly outperforms the paragraph-based one, as well as the other systems that participated in the BB Shared Task 2013.

## Background

### Introduction

Identifying and characterizing the habitats where bacteria live (i.e. bacteria biotopes) is crucial for gaining a better understanding of bacterial infections, which in turn can lead to the development of novel disease prevention, prediction, and treatment methods. Besides health sciences, information about the relations of bacteria with their environments is also important for research areas such as microbiology, agronomy, and food processing and preservation. One of the challenges that researchers in these areas face is the absence of a comprehensive database that stores the relationships among bacteria and their habitats in a structured format. Most of the bacteria habitat information is only available in an unstructured textual format in electronic resources such as scientific publications and web pages of bacteria sequencing projects [1]. For instance, even a limited search in PubMed for "*bacteria AND (habitat OR localization OR environment)*", which probably barely covers all relevant documents, returns 177, 000 documents (Search date: January 29, 2014). This illustrates the difficulty of manual curation for developing a comprehensive database that stores and provides easy access to information about bacteria and their habitats. An important step towards the creation and population of such a database is developing text mining methods to automatically recognize and normalize mentions of bacteria and habitats in text, as well to identify the relations among them.

The Bacteria Biotope (BB) Task in the BioNLP Shared Task 2013 addressed the problems of identifying locations where bacteria live and semantically annotating them using an ontology [1-3]. Unlike most previous biomedical information extraction challenges which target extracting information from publications in PubMed (e.g. [4-6]), the documents targeted in the BB task are scientific web pages. In addition these documents are richer in terms of both the number and the variety of habitats, compared to the ones in PubMed [1].

**Figure 1:**
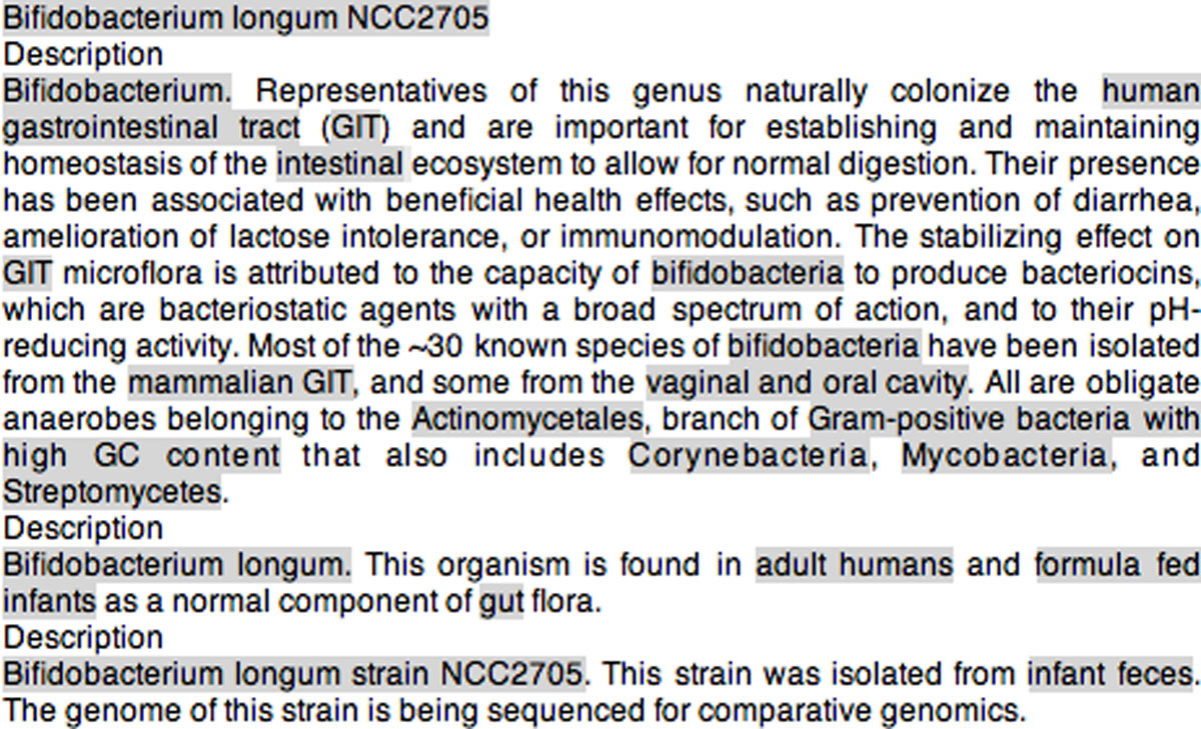
**Sample text**. A sample input file containing bacteria and habitat entities.

The BB task consisted of three sub-tasks. **Sub-task 1 **involved the recognition of habitat names in text and their categorization with concepts from the OntoBiotope (MBTO) Ontology [7]. Figure 1 shows a sample text file from the training set provided by the organizers. The bacteria and habitat entities are shown in bold. For instance, *"Bifidobacterium" *is a bacteria entity, whereas *"human" *and *"human gastrointestinal tract" *are habitat entities. The concept that is associated with the *"human gastrointestinal tract" *habitat in the OntoBiotope ontology is *"digestive tract"*, and the one associated with the *"human" *habitat is *"human"*.

Given the names, types (i.e. *Bacteria, Habitat, Geographical*), and positions of the entities in text the goal of **Sub-task 2 **was to extract the localization relations between bacteria and habitat (i.e. *Habitat, Geographical*) pairs, as well as PartOf relations between habitat pairs. A PartOf relation between a pair of habitats holds if one of them is a living organism (called host), and the other one is part of this organism (called host part). The relation between *"Bifidobacterium" *and *"human gastrointestinal tract"*, as well as the one between *"Bifidobacterium" *and *"human" *are among the localization relations described in the text shown in Figure 1. The relation between the host *"human" *and the host part *"human gastrointestinal tract" *is one of the PartOf relations described in Figure 1. One of the challenges in the relation extraction task is the high frequency of bacteria anaphors and relations that cross sentence boundaries.

**Sub-task 3 **was similar to Sub-task 2. The only difference in Sub-task 3 was that the gold standard entity annotations were not given to the participants. In other words, the participants were also expected to detect the bacteria and habitat entities.

In this paper, we describe our submissions to Sub-task 1 (Entity Detection and Categorization) and Sub-task 2 (Localization Relation Extraction) in the BB Task of the BioNLP Shared Task 2013 [8], as well as the new methods that we developed and the improvements that we obtained after the official evaluation. We propose a linguistically-oriented rule-based approach for entity detection and categorization. Our approach utilizes the shallow syntactic analysis of the text including sentence segmentation, tokenization, lemmatization, part-of-speech tagging, and shallow (partial) parsing. Manually designed syntactic rules that are based on the noun phrases and the part-of-speech tags of the words in the sentences are used to recognize the habitat entities and map them to the corresponding concepts in the OntoBiotope ontology. Our approach also tackles the problem of handling discontinuous entities such as the two distinct entities *"nasal cavity" *and *"oral cavity" *in the phrase *"nasal and oral cavity"*.

As improvements to the Sub-task 1 system, we investigate expanding the OntoBiotope ontology using the training set and extending the noun phrases with their modifiers including the ones that are attached with the prepositions *in, of*, and *with *(e.g. *"infected child in Germany"*). In this article, we also introduce and compare two different approaches for relation extraction. Our original submission to Sub-task 2 was based on the first approach, which assumes that discourse changes with a new paragraph, and associates the habitat entities with the first bacteria mention in the paragraph. After the official shared task evaluation, we developed a novel sentence-based approach that incorporates an anaphora resolution module to handle bacteria coreferences.

## Related work

Due to the continued rapid increase in the number of scientific articles published in the biomedical domain, it has become difficult for scientists to reach and make use of the knowledge contained in the biomedical scientific literature. Therefore, developing text mining systems for automatically extracting the biologically useful information from biomedical text has become crucial [9]. A number of shared tasks including the LLL and BioCreative Challenges, as well as the BioNLP Shared Tasks have been conducted, which have facilitated research in biomedical text mining [4,6,10,11]. Most of these shared tasks addressed the problems of relation or event extraction among biomolecular entities such as proteins and genes.

The Bacteria Biotope Task is the first shared task targeting the extraction of information about bacteria and their habitats. This task was first conducted in the BioNLP Shared Task 2011 [12-14]. Among the three teams that participated in the Bacteria Biotope Task 2011 [12,13], Bibliome INRA [15] obtained the best F-score performance (45%) on the task of identifying habitat entities. They made use of resources including a list of Agrovoc geographical names [16], the NCBI Taxonomy [17], as well as an ontology for location types, and developed a system that is based on ontology-based reasoning and linguistic features. UTurku [18] developed a generic machine learning based system that can be used for all the main tasks in the BioNLP Shared Task 2011 with minor modifications. They incorporated this generic system with additional named entity recognition patterns and external resources for identifying the named entities and their types in the Bacteria Biotope Task. JAIST [19] also used a machine learning approach based on Conditional Random Fields (CRFs) [20] for this task. UTurku and JAIST adapted machine learning approaches for detecting the Localization and Part-of relations among bacteria and habitats. On the other hand, Bibliome developed a rule-based system based on the co-occurrence of entities with a trigger word in the same sentence. Only the Bibliome team performed coreference resolution. UTurku's system was based on sentence level processing, whereas JAIST's system was based on paragraph level processing. Therefore, Uturku's system was most affected from not performing coreference resolution [12,13].

The Bacteria Biotope (BB) Task in the BioNLP 2013 Shared Task gave another opportunity to scientists to address the task of extracting information about bacteria and their habitats from text and evaluate their approaches on a common platform [1,2]. This task maintained the primary objective of the 2011 edition of the BB task of extracting bacteria and localization relations. In addition, it introduced a new task that targeted a more fine-grained categorization (i.e. normalization) of habitat entities through the OntoBiotope ontology. Five teams participated in the 2013 edition of the BB Task [1,2]. For Sub-task 1 the systems were ranked according to their slot error rates (SER). The first three systems obtained similar SER performances for this Sub-task despite their different approaches to the problem [1,2]. The LIPN system [21] based on a machine learning approach achieved the best SER score (66%) in Sub-task 1. The best F-score (42%) for Sub-task 2 was obtained by the TEES 2.1 system [22], which used multi-step Support Vector Machine classification. TEES 2.1 obtained the best F-score of 14% and a relaxed score of 49% in Sub-task 3 as well. TEES 2.1 is a generalized tool for relation extraction that was implemented to apply to many tasks in the BioNLP Shared Task. It did not tackle Sub-task 1 of the BB task that aimed at identifying the habitat entities and assigning them to the corresponding OntoBiotope ontology concepts. The IRISA system used a machine learning approach based on the k-Nearest Neighbor (kNN) method and obtained a SER score of 93% in Sub-task 1, and ranked second with an F-score of 40% in Sub-task 2 [23]. LIMSI [24] was the only team that participated in all three BB sub-tasks. They used a method based on Conditional Random Fields [25] for the official submissions, while they utilized Maximum Entropy models for later improvements. They utilized various additional resources such as NCBI taxonomy for the detection of bacteria names, the Cocoa [26] annotations for the categorization of bacteria, habitat, and geographical entities, and OntoBiotope Ontology for the identification of habitat names. They obtained a SER value of 68% in the official submissions for Sub-task 1. Their results for Sub-task 2 and Sub-task 3 were relatively lower. We participated in Sub-task 1 and Sub-task 2 of the BB Task 2013. Our system Boun ranked second in Sub-task 1 with a SER score of 68% and third in Sub-task 2 with an F-score of 27% in the official evaluation [8]. The Sub-task 1 module of the Boun system utilized the shallow syntactic analysis of the text and linguistically-motivated rules. The Sub-task 2 system submitted to the official evaluation was based on a paragraph-based relation extraction approach, where the habitat entities were assumed to be related to the bacteria entity that occur first in the paragraph. The improvements that we developed after the shared task can be summarized as extending the OntoBiotope ontology using the training set, and developing a novel method for Sub-task 2. This method operates on a sentence basis. In order to handle relations that span multiple sentences a new anaphora resolution approach for the bacteria biotopes domain has been developed as well. These improvements led to state-of-the-art results in Sub-task 2. The extended system *Boun 2 *obtained 68% SER on Sub-task 1, and 53% F-score on Sub-task 2. The details of our official submission as well as the improvements developed after the shared task are described in the following sections.

Sub-task 1 of the BB Task is related to the general problem of named entity recognition (NER) and automatic semantic annotation by ontologies. Rule-based approaches (e.g. [27]), as well as machine-learning based methods (e.g. [28,29]) have been developed for biomedical NER. While state-of-the-art NER systems for proteins and genes achieve performance levels that enable their use in practice, the problem of recognizing bacteria habitat names in text has not been tackled prior to the 2011 and 2013 editions of the BB Task, and there is still a lot of room for improvement. Different approaches for the semantic annotation of entities using ontologies have been proposed in the literature. Our approach is related to rule-based methods that make use of the syntactic and semantic analysis of the terms [30,31]. A problem related to ontology-based semantic tagging has also recently been addressed in the Biocreative III Interaction Method Task (IMT) [32]. The goal was to identify the interaction methods in the articles and normalize them through the PSI-MI ontology [33]. The best performing systems in the shared task employed machine learning methods [34,35]. However, they formulated the problem as classifying the entire articles to the ontology concepts, and did not address the problem of identifying the boundaries of the named entities. The relatively smaller training set size in the BB Task and the large number of classes (i.e. 1700 concepts) pose challenges for machine learning based classifiers in this domain.

Sub-task 2 of the BB Task is related to the general problem of relation extraction. A number of different methods including entity co-occurrence based approaches [36,37] and pattern matching based approaches [38-40] have been developed for extracting relations among biomedical entities including genes, proteins, drugs, and diseases. The state-of-the-art techniques for biomedical relation extraction are in general based on using the syntactic analyses of the sentences, usually in conjunction with machine learning methods [41-45]. Most relation extraction systems operate on a sentence-level. The underlying assumption is that the majority of the relations are contained within a single sentence. This assumption holds for some domains. For example, it has been shown that only 5% of the relations in the Genia event corpus [11] span multiple sentences [46]. However, a challenge in the Bacteria Biotopes domain is the vast amount of relations that span multiple sentences and the abundance of bacteria anaphora in the text. Despite this fact, only one of the systems that participated in the BB Shared task 2011 tackled the anaphora resolution problem in this domain [15], and none of the systems in the BB Task 2013 included anaphora resolutions modules [1].

## Methods

### Entity boundary detection and ontology categorization

**Figure 2:**
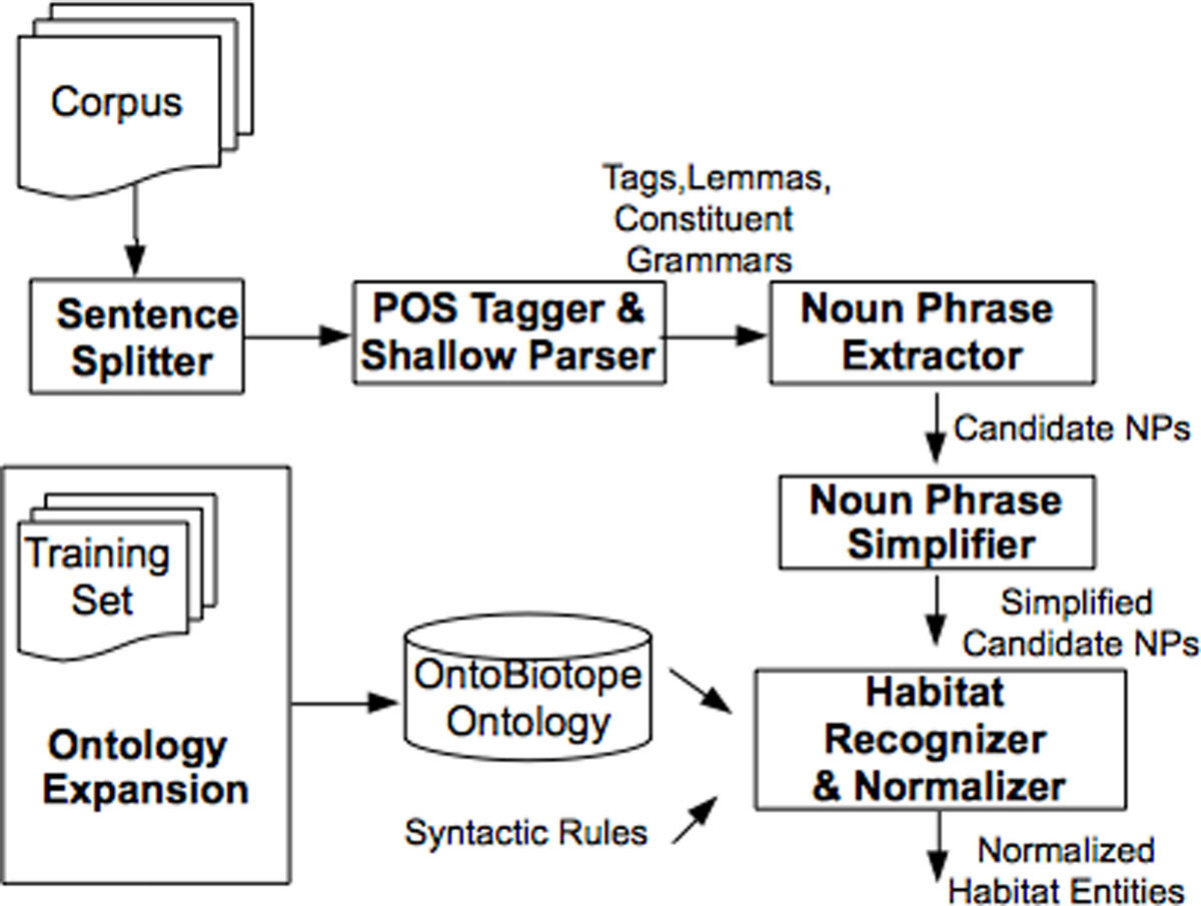
**Workflow of the Sub-task 1 System**.

We developed a linguistically motivated rule-based system for Sub-task 1 (Entity Detection and Categorization), the workflow of which is displayed in Figure 2. The input text is first pre-processed by splitting into sentences and performing shallow syntactic analysis including POS tagging, lemmatization, and partial parsing. Based on our observation in the training set, we assume that most habitat entities are noun phrases. Before normalizing through the OntoBiotope ontology, the candidate habitat entities are identified by extracting and simplifying the noun phrases in the sentences. In addition, the OntoBiotope ontology is expanded by using the training set. We also investigate handling discontinuous entities and entity modifiers. The details of our approach are described in the following subsections.

#### Preprocessing

**Figure 3:**
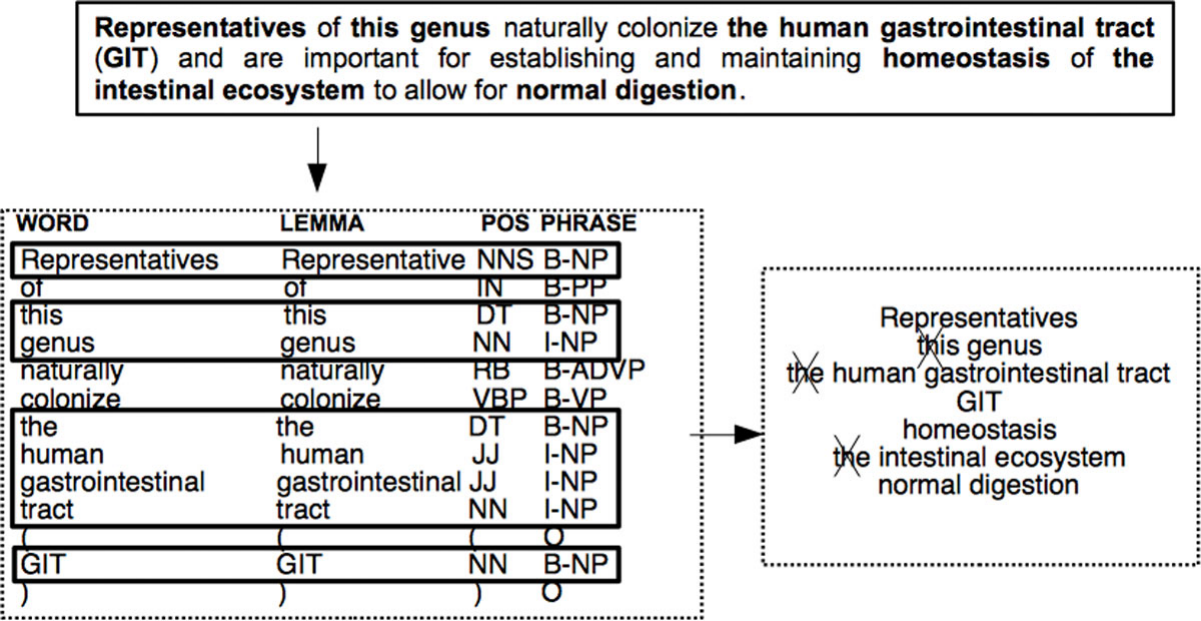
**Sample output of the preprocessing, and the noun phrase extractor and simplifier**.

In the preprocessing step, we used the Genia Sentence Splitter (GeniaSS) [47] to segment the text into sentences and the Genia Tagger [28,48] to obtain the shallow linguistic features of these sentences including the POS tags, the lemmas, and the constituent categories of the words. Figure 3 shows a sample sentence and the output obtained by the preprocessing module (on the left-hand side of the figure). These shallow syntactic analysis results are then used in the following steps of our system to extract and simplify the noun phrases (as shown on the right-hand side of Figure 3), as well as to map them to the OntoBiotope ontology.

#### Ontology expansion from the training data

In this step, the annotated training data set is used to expand the OntoBiotope ontology. If a term in the training set is labeled with an OntoBiotope ontology concept, it is included in the ontology as a synonym of that concept, unless it is already defined as a name or as a synonym of that concept. For example, the ontology concept with ID *MBTO:00001875 *has the name *"mummy tissue" *in the ontology. This entry does not have any synonyms. However, in the training set the term *"tissues of ancient mummies" *is labeled with this concept. Therefore, *"tissues of ancient mummies" *is added as a synonym of the *"mummy tissue" *concept in the ontology.

#### Noun phrase extraction and simplification

In the noun phrase extraction and simplification step, first, the noun phrases are extracted based on the constituent categories of the words identified by the Genia Tagger. Next, the extracted noun phrases are simplified by removing the words that do not contain informative information regarding bacteria habitats. The non-informative words are identified based on their POS tags. For instance, determiners and possessive pronouns are non-informative and thus, are not included in the boundaries of the habitat entities. Consider the noun phrases "*the **mummy tissue***" and "*its **small intestine***". The simplified noun phrases are obtained by removing the determiner *"the" *from the first noun phrase and the possessive pronoun *"its" *from the second noun phrase. Thus, the simplified noun phases are "*mummy tissue*" and "*small intestine*", respectively. The preprocessing, noun phrase extraction and simplification processes are illustrated in Figure 3 for a sample sentence.

#### Discontinuous entity handling

Some habitat entity spans in text may be discontinuous. For example, the phrase *"ground and surface water" *contains two overlapping entities, namely *"ground water" *and *"surface water" *[1]. Our system includes a mechanism to handle discontinuous entities, which are represented with noun phrases containing the conjunction *"and"*. Such noun phrases are split into two sub-phrases from the conjunction *"and"*. If the two sub-phrases map to two concepts in the OntoBiotope ontology, which have the same direct ancestor represented with a common is-a relation, then the habitats are identified according to the structure of the noun phrase as follows. Each sub-phrase is considered to be a separate habitat entity, if both of the sub-phrases consist of single words tagged as nouns. Otherwise, the two sub-phrases constituting the noun phrase are identified as a single habitat entity. On the other hand, if the mapped two concepts in the OntoBiotope ontology don't have a common direct ancestor, then the corresponding two sub-phrases are considered to be two separate habitat entities. Our approach for discontinuous entity handling is described in more detail below through the example phrases *"pharyngeal and gut mucosa" , "iron-rich and wet environment", "plants and animals"*, and *"mouse and cheese"*.

**Figure 4:**
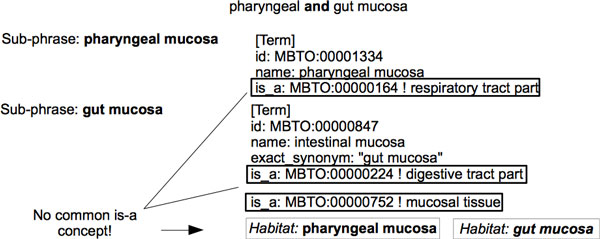
**Discontinuous entity handling for the sample phrase *"pharyngeal and gut mucosa"***.

**Figure 5:**
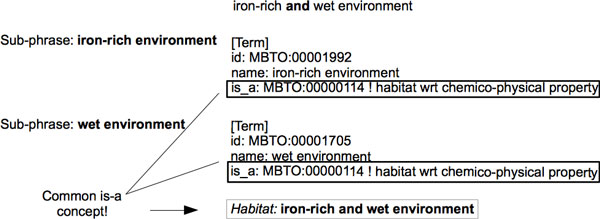
**Continuous entity handling for the sample phrase *"iron-rich and wet environment"***.

• Given the phrase *"pharyngeal and gut mucosa"*, the two generated sub-phrases are *"pharyngeal mucosa" *and *"gut mucosa"*. The direct ancestor of *"pharyngeal mucosa" *in the OntoBiotope ontology is *"respiratory tract part"*, whereas the direct ancestors of *"gut mucosa" *are *"digestive tract part" *and *"mucosal tissue"*. Since the OntoBiotope ontology concepts corresponding to the two sub-phrases don't have a common direct ancestor, these sub-phrases are identified as two different habitat entities, namely *"pharyngeal mucosa" *and *"gut mucosa" *(See Figure 4).

• Given the phrase *"iron-rich and wet environment"*, the two generated sub-phrases are *"iron-rich environment" *and *"wet environment"*. The two concepts corresponding to these sub-phrases in the OntoBiotope ontology have a common direct ancestor, which is *"habitat wrt chemico-physical property"*. Therefore, a single habitat entity (i.e., *"iron-rich and wet environment"*) corresponding to the entire noun phrase is generated (See Figure 5).

• Given the phrase *"plants and animals"*, the two generated sub-phrases are *"plants" *and *"animals"*. The two concepts corresponding to these sub-phrases in the OntoBiotope ontology have the *"eukaryote host" *direct ancestor. However, since both sub-phrases consist of single words, which are tagged as nouns, two different habitat entities are identified, namely *"plants" *and *"animals"*.

• Given the phrase *"mouse and cheese"*, the two generated sub-phrases are *"mouse" *and *"cheese"*. The concepts corresponding to these sub-phrases in the OntoBiotope ontology don't have a common direct ancestor. Therefore, two different habitat entities, namely *"mouse" *and *"cheese"*, are identified.

#### Entity modifier handling

The data set for the Bacteria Biotopes shared task has been annotated by including the modifiers that describe the habitats in the boundaries of the habitat entities [1]. Consider the phrase *"infected infant in Germany"*. The ontology concept that this phrase is mapped to is *"infant" *(*MBTO:00000778*). However, the boundary of the habitat entity is the entire phrase, namely *"infected infant in Germany"*. The shallow parser labels *"infected infant" *and *"Germany" *as two separate noun phrases and *"in" *is labeled as a preposition. After the official evaluation, our system has been extended to handle the habitat entities that contain modifiers. If a noun phrase (*NP*) is followed by a preposition (*prep*) and then by another noun phrase, the entire *NP prep NP *sequence is identified by the noun phrase extraction and simplification module as a candidate habitat entity. Besides the prepositional phrases that contain *"in"*, the ones that contain *"of" (e.g. "respiratory tract of animals"*) and *"with" *(e.g. *"2-year-old girl with tick-bourne relapsing fever"*) are also handled using the same approach. However, as discussed in the Results section this extension degraded the performance of the system.

#### Ontology mapping

To identify whether the phrases extracted in the previous steps correspond to habitat entities and to determine the boundaries of the habitat entities, exact or partial matching against the names and synonyms of the concepts in the OntoBiotope ontology is performed.

**Figure 6:**
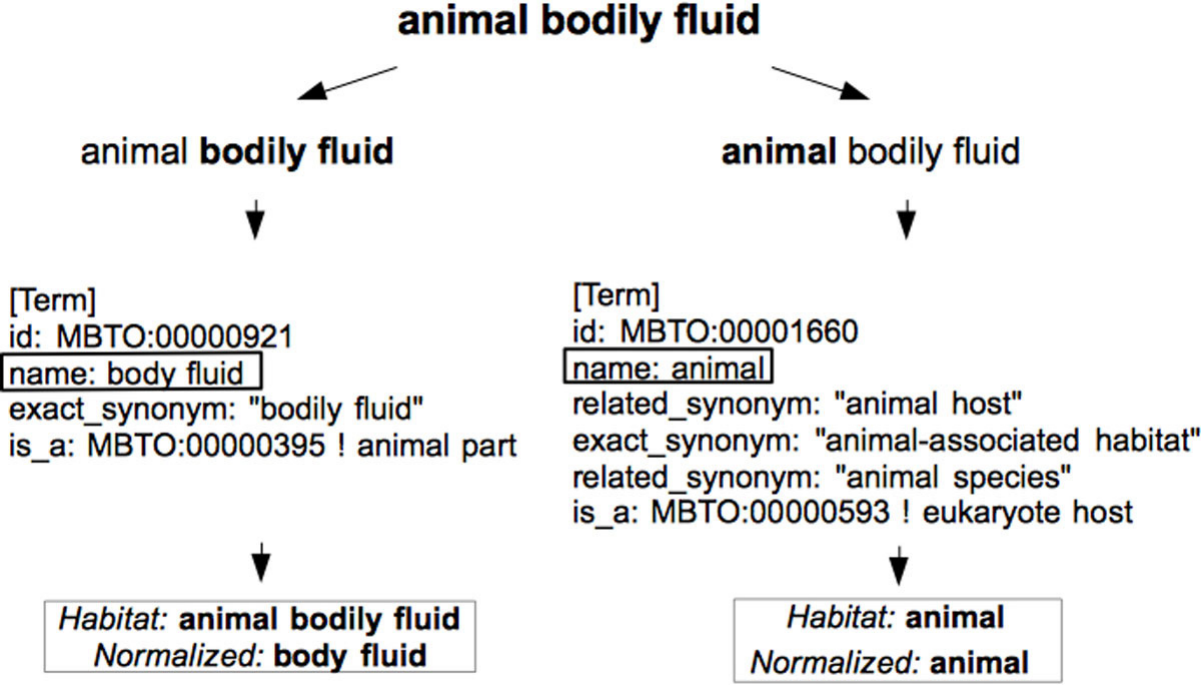
**Ontology mapping example**.

Consider the extracted noun phrase *"the animal bodily fluid"*. In the noun phrase simplification step, this phrase is simplified as *"animal bodily fluid"*, which is searched against the OntoBiotope ontology for exact or partial matches. As shown in Figure 6, this candidate phrase is mapped to two ontology concepts. It is mapped to the concept *"body fluid" *due to the partial match with the *exact synonym: "bodily fluid"*. Similarly, it is mapped to the concept *"animal" *due to the partial match with the concept *name: animal*.

The boundaries of the habitat entities are identified by using the following manually designed syntactic rules.

• If there is an exact match between an ontology concept and a candidate phrase, the candidate phrase is identified as a habitat entity and the entity boundary is set as the boundary of the candidate phrase.

• If there is a partial match between a candidate phrase and an ontology concept such that the match begins from the first word of the candidate phase, but does not cover the entire phrase, the matching sub-phrase of the candidate phrase is identified as a habitat entity and the entity boundary is set as the boundary of the matching sub-phrase. For instance, as shown in Figure 6, the first word of the candidate phrase *"animal bodily fluid" *matches with the *name: "animal" *of the ontology concept for *animal*. Therefore, the habitat entity *"animal" *is identified and normalized to the ontology concept with *MBTO:00001660*.

• If there is a partial match between a candidate phrase and an ontology concept such that the match does not begin from the first word of the candidate phrase, the candidate phrase is identified as a habitat entity and the boundary of the entity is set as the boundary of the phrase. For instance, in Figure 6, the candidate phrase *"animal bodily fluid" *matches with the *exact synonym: "bodily fluid" *of the ontology concept for *body fluid*, starting with the second word of the candidate phrase. Therefore, the entire candidate phase *"animal bodily fluid" *is identified as a habitat entity and normalized to the ontology concept with *MBTO:00000921*.

In order to match the different inflected forms of the habitat names such as matching the habitat name *"animal" *against its plural form *"animals"*, we performed lemmatization on the candidate phrases by using the Genia Tagger, and applied the same methodology that is explained above not only to the surface forms of the candidate phrases, but also to their lemmatized forms.

### Relation extraction

In this section we describe the systems that we developed for extracting bacteria localization and habitat PartOf relations.

#### Localization relation extraction

One of the two types of relations that have to be extracted for Sub-task 2 is the localization relations between bacteria and habitat entities. For example, the following excerpt from an input text file *"Bordetella. This group of organisms is capable of invading the respiratory tract of animals and causing severe diseases."*, contains information about *"Bordetella" *bacteria that lives in the *"respiratory tract of animals"*. Therefore, there are localization relations between the *"Bordetella" *bacteria entity and the *"respiratory tract of animals" *and *"animals" *habitat entities, which must be extracted automatically.

In order to extract localization relations between bacteria and habitat entities, we propose two different systems. The paragraph-based system is the official system which was submitted to BioNLP Shared Task 2013 [8]. The sentence-based system with the anaphora resolution module was developed after the official evaluation, and is a novel contribution of this paper. In the following subsections, each system is explained in detail.

#### Paragraph-based system

This system is based on the assumption that the bacteria name that occurs first in a paragraph is the topic of that paragraph. Therefore, after identifying the bacteria and habitat entities in a paragraph, the bacterium that appears first in the paragraph is associated with all habitat entities in that paragraph. If this bacterium entity occurs earlier in the document as well, then its first occurrence in the document is associated with the habitat entities in the paragraph. A special rule is applied to bacteria names that contain the term *"strain"*. In this case, the habitat entities are associated with the first occurrence of the corresponding bacterium name that does not contain the *"strain" *term. For example, in a paragraph that starts with the sentence *"Bordetella petrii strain DSM12804 was initially isolated from river sediment"*, a relation is set between the habitat entity *"river sediment" *and the bacterium entity *"Bordetella petrii DSM12804" *that occurs earlier in the document, instead of *"Bordetella petrii strain DSM12804"*, which is the first bacterium name in the given paragraph.

#### Sentence-based system

**Figure 7:**
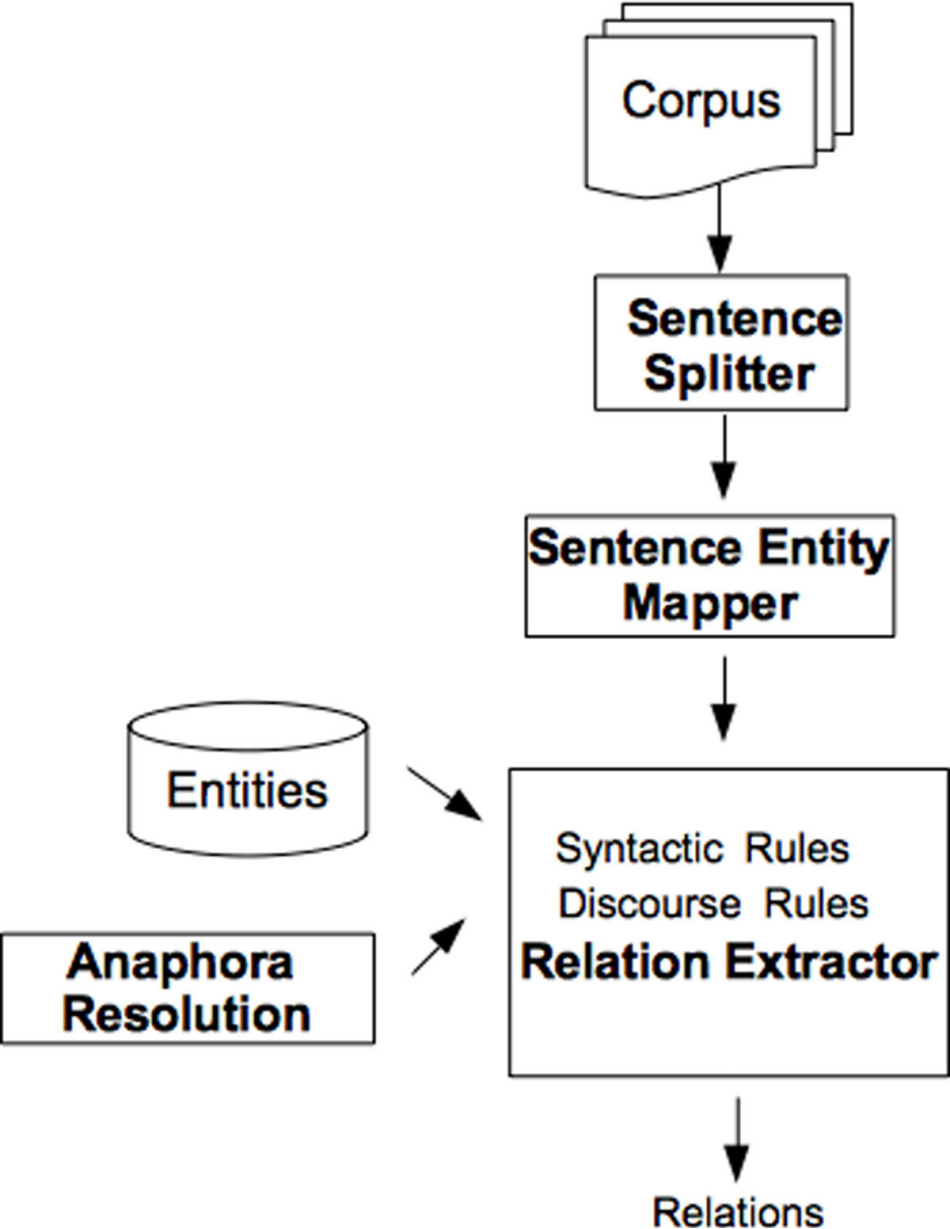
**Workflow of the Sentence-based Sub-task 2 System**.

The workflow of the sentence-based system is shown in Figure 7. This system operates on a sentence-basis and performs a more fine-grained analysis of the text compared to the paragraph-based system. First, the text is segmented into sentences. Then, the bacteria and habitat entities that occur in the given sentence are identified. The assumption is that there is a relation between bacteria and habitat entities that occur in the same sentence, if there is a specific bacteria name in the considered sentence. For example, *"Bordetella petrii DSM12804" *is a specific bacteria name, whereas the terms *"bacteria" *and *"bacterium" *are not specific bacteria names, even though they are tagged as bacteria entities in the text documents.

#### Anaphora resolution

One of the challenges for extracting bacteria localization relations is that the corpus contains a large number of anaphora. In general, each document in the corpus is about a specific bacterium species [1]. After an explicit mention of the name of this species in a sentence, it is often referred to by using anaphors in the subsequent sentences. Therefore, several localization relations span multiple sentences. To tackle this problem we developed an anaphora resolution module and integrated it with the sentence-based localization relation extraction system. The anaphora resolution module detects sentences that do not include any bacteria entities, but contain coreferences to bacteria entities. There are three types of anaphoric expressions which are handled in different ways by our system:

*Anaphora type 1: *We compiled a keyword list consisting of 23 anaphoric expressions such as *"the bacterium", "this organism", "this species", "this genus"*, and *"this group of organisms" *by manually analyzing the training set. If a sentence does not contain a bacteria name, but contains an anaphoric expression included in the keyword list, the antecedent of the anaphor is set as the first bacteria name that occurs in the previous sentence. Then, localization relations are identified between the habitats in the sentence and the detected antecedent of the anaphoric expression. For example, although the sentence *"This bacterium is highly infectious, and can be spread through the contact with the infected animal products or through the air," *does not include any explicit bacteria entity names, it describes localization relations between the bacteria anaphor *"This bacterium" *and the habitats *"animal products" *and *"air"*. In this case, the anaphora resolution module looks at the previous sentence, which is *"Brucella canis." *and assigns the habitat entities to this bacteria entity. If there is no bacteria name in the previous sentence, then the first bacteria entity in the document is assigned to the habitat entities, since in general each document is about a specific bacterium species, and the mention of this species occurs first in the document.

*Anaphora type 2: *If there is no specific bacteria name in the given sentence, but the sentence begins with the anaphoric pronoun *"it"*, then our system looks at the previous sentence and a localization relation is set with the first bacteria in the previous sentence and the habitats in the given sentence. For example, given the sentence *"It was isolated from Ixodes scapularis in 1982,"*, our system looks at the previous sentence *"Borrelia burgdorferi" *and sets a localization relation between the *"Borrelia burgdorferi" *bacteria entity and the *"Ixodes scapularis" *habitat entity.

*Anaphora type 3: *If a sentence begins with the *"This strain" *anaphoric expression, then similarly to the paragraph-based system, the bacteria entity that occurs first in the document is assigned as the antecedent of the anaphor. Consequently, the habitat entities in the sentence are assigned to this antecedent.

#### PartOf relation extraction

PartOf relations between habitat entities is the second relation type targeted in the BB Shared Task. For example, in the sentence *"This strain was isolated from infant feces"*, the habitat entity *"infant feces" *is a part of the habitat *"infant"*. For habitat PartOf relation extraction we introduce a shallow syntactic analysis dependent rule-based approach. The first rule with the preposition *"of" *was developed for the official shared task submission. The remaining rules were developed after the shared task. Our rules are based on the assumption that a habitat is likely to be a part of another habitat, if the mention of the second habitat in text contains the mention of the first habitat, and in addition the syntactic rules described below are met.

*Syntax rule 1: *If one habitat contains the other one, and the second habitat follows one of the prepositions *"of", "in", "from"*, then the relation that the first habitat is PartOf the second habitat is extracted. For example, the habitat mention *"rhizosphere of plants" *contains the *"plants" *habitat mention. Since the first habitat phrase contains the preposition *"of"*, and the second habitat phrase *"plants" *occurs right after this preposition, the relation *"rhizosphere of plants" *is PartOf *"plants" *is extracted. As another example, the habitat mention *"oral cavity in humans" *contains the *"humans" *habitat mention. Since the first habitat mention contains the preposition *"in"*, and the second habitat mention *"humans" *follows this preposition, the relation *"oral cavity in humans" *is PartOf *"humans" *is extracted. Finally, *"skin lesion from a Lyme disease patient in Europe" *and *"Lyme disease patient in Europe" *are overlapping habitat entities, one of which contains *"from"*, which is succeeded by the second habitat mention. Then, the relation *"skin lesion from a Lyme disease patient in Europe" *is PartOf *"Lyme disease patient in Europe" *is extracted.

*Syntax rule 2: *If two habitat mentions overlap in text like in the example *"Aeschynomene stem nodule" *and *"Aeschynomene"*, by looking at their positions we infer a PartOf relation between them. For example, *"Aeschynomene stem nodule" *is PartOf *"Aeschynomene"*.

## Results and discussion

### Data set

The training, development, and test sets provided by the BB shared task organizers contain 52, 26, and 26 documents, respectively. The gold standard annotations for the training and development sets were provided to the participants, whereas the evaluations on the test set were performed by using the online evaluation tool released by the shared task organizers. The documents in the corpus consist of web pages obtained from a number of web sites such as from the web sites of bacteria sequencing projects or MicrobeWiki [1].

### Evaluation metrics

The main evaluation metric used for Sub-task 1 is Slot Error Rate *(SER) *[1]. Lower SER values denote better performance, since SER is an error measure. The computation of SER is shown in Equation 1, where *S, D*, and *I *correspond to the number of substitutions, deletions, and insertions, respectively. *N *is the total number of habitats in the reference. If a reference entity does not match exactly or partially with any of the predicted entities, then this corresponds to a deletion, i.e., to a false negative. On the other hand, if a predicted entity does not match exactly or partially with any of the reference entities, then this corresponds to an insertion, i.e., to a false positive. *D *and *I *are the numbers of false negatives and false positives, respectively.

(1) $$SER=\frac{S+D+I}{N}$$

The computation of *S *is shown in Equation 2.

(2) S=1-M

Here, *M *is the similarity between two entities. It is computed by using Equation 3. The more similar two entities are, the lower their substitution score is.

(3) M=J⋅W

*J *in Equation 3 is the Jaccard coefficient similarity between the predicted and reference entities [13]. If the boundary of the predicted entity is exactly the same as the boundary of the reference entity, then *J *equals 1 for the pair. The less the entities overlap, the lower the value of *J *is. *W *is a parameter that measures the similarity between the ontology concepts of the reference and the predicted entities [49]. It is based on the Jaccard coefficient of the sets of ancestors corresponding to the reference and predicted entities. The value of *W *is 1 if the predicted entity and the reference entity are assigned to the same concept in the ontology, and it is less than 1 if they are assigned to different entities. The higher the value of *W*, the more similar the two concepts are to each other.

The evaluation metrics used for Sub-task 2 are precision, recall, and f-score. The details of the evaluation metrics and the official evaluation results are available in [1]. In the following subsections, the results of the system (*Boun*) with which we participated in the BB shared task and the results of the improved system (*Boun 2*) developed after the official evaluation are presented.

### Results for sub-task 1

Table 1 shows the detailed results obtained on the test set for Sub-task 1 by the *Boun *and *Boun 2 *systems. The workflows of both systems are the same (Figure 2), except the ontology expansion module, which is only available in the *Boun 2 *system and a new additional rule for discontinuous entity handling. Both systems perform discontinuous entity handling, and neither of them perform entity modifier handling. These results show that expanding the OntoBiotope ontology using the training set, did not lead to improvements in the performance of the system. Since the concepts in the ontology are enriched by including more synonyms, more entities in the test set are matched to their concepts in the ontology. This resulted in a lower number of false negatives (i.e., lower D) and higher number of matches, which leads to higher recall and F-score values. While the SER value does not change, due to the increase in the number of false positives (i.e., insertions), the precision of the system decreases.

**Table 1:** Detailed results on the test set for Sub-task 1 *(Entity Boundary Detection & Ontology Categorization)*

Evaluation Metrics	Boun	Boun 2
**S**	112.70	115.24
**I**	141	158
**D**	89	74
**M**	305.30	317.75
**SER**	0.68	0.68
**Recall**	0.60	0.63
**Precision**	0.59	0.57
**F-score**	0.59	0.60

Table 2 presents a comparison of the results obtained by the *Boun *and *Boun 2 *systems, and the other systems that participated in the Bacteria Biotope 2013 Sub-task 1. The *Boun *system that we submitted to the official evaluation ranked second among four systems in terms of the SER evaluation metric. The *Boun 2 *system also achieves a SER value (68%) which is close to the *LIPN *system that ranked first in the shared task. In addition, the precision and recall values of the *Boun *and *Boun 2 *systems are relatively more balanced compared to the other systems except the *LIPN *system.

**Table 2:** Comparison with the other systems that participated in the BB Sub-task 1 *(Entity Boundary Detection & Ontology Categorization)*.

System	SER	Recall	Precision	F-score
**LIPN**	0.66	0.61	0.61	0.61
**Boun**	0.68	0.60	0.59	0.59
**LIMSI**	0.68	0.35	0.62	0.44
**Boun 2**	0.68	0.63	0.57	0.60
**IRISA**	0.93	0.72	0.48	0.57

The results obtained on the test set are reported.

Table 3 shows the effect of the discontinuous entity handling (DEH) module. The first column displays the results obtained by the *Boun 2 *system, whereas the second column shows the results obtained by removing the discontinuous entity handling module from the system. These results demonstrate that performing discontinuous entity handling leads to a lower SER value, i.e., to a better performance on the training and development sets. On the other hand, the discontinuous entity handling module does not make any particular change in the SER value of the system on the test set.

**Table 3:** Effect of discontinuous entity handling (DEH).

	Boun 2	Boun 2 - DEH
**SER Train**	0.66	0.67
**SER Dev**	0.67	0.68
**SER Test**	0.68	0.68

The results are reported on the training, development, and test sets.

Table 4 demonstrates the effect of the entity modifier handling module. The first row presents the results obtained by the *Boun 2 *system, whereas the subsequent rows show the results obtained by extending the *Boun 2 *system by including a mechanism to handle the modifiers attached to the noun phrases with the prepositions *in, of*, and *with*. Due to the fact that the SER values obtained by the system with the entity modifier handling module are not lower than the *Boun 2 *system for the training and development sets, this module is not included in the final system. The results reveal that the introduced entity modifier handling approach reduces the performance of the system, due to the prepositional phrase attachment ambiguity problem. For example, consider the sentence *"This species was isolated from a Lyme disease patient in Europe"*. Our entity modifier handling approach correctly identifies the habitat *"Lyme disease patient in Europe" *by extending the *"Lyme disease patient" *noun phrase with its modifier *"in Europe"*. However, given the sentence *"This species was isolated from a Lyme disease patient in 1993"*, the habitat is incorrectly identified as *"Lyme disease patient in 1993"*. The prepositional phrase *"in 1993" *is incorrectly attached to the noun phrase, whereas it should have been attached to the verb *"isolated"*. Handling complex nominals and resolving such prepositional phrase attachment problems can be possible by using a full syntactic parser, rather than a partial parser.

**Table 4:** Effect of entity modifier handling.

	SER Train	SER Dev	SER Test
**Boun 2**	0.66	0.67	0.68
**Boun 2 + in**	0.68	0.67	0.70
**Boun 2 + of**	0.72	0.72	0.72
**Boun 2 + with**	0.67	0.67	0.68

The results are reported on the training, development, and test sets.

### Results for sub-task 2

This section provides the evaluation results obtained by the paragraph-based system (*Boun*) with which we participated in the BB Shared Task Sub-task 2 (Localization and PartOf Event Extraction), as well as the newly developed sentence-based system with the anaphora resolution module (*Boun 2*). Table 5 presents a comparison of the *Boun *and *Boun 2 *systems with each other. The results demonstrate that the *Boun 2 *system performs significantly better than the *Boun *system.

**Table 5:** Results of BB Sub-task 2 *(Localization and PartOf Event Extraction)*.

System	Type	Recall	Precision	F-score
**Boun 2**	Localization	0.61	0.54	0.57
	PartOf	0.20	0.32	0.25

**Boun**	Localization	0.23	0.38	0.29
	PartOf	0.15	0.40	0.22

The results obtained on the test set are reported

Table 6 presents a comparison of the *Boun *and *Boun 2 *systems with the other systems that participated in the shared task. According to the official results, the *Boun *system ranked third among the four systems that participated in the event detection task. The new *Boun 2 *system achieves 53% F-score on the test set, which is significantly higher than the 27% F-score obtained by the *Boun *system. The F-score of the *Boun 2 *system is even higher than the F-score of the system that ranked first in the official evaluation.

**Table 6:** Comparison with the other systems that participated in the BB Sub-task 2 *(Localization and PartOf Event Extraction)*.

System	Recall	Precision	F-score
**Boun 2**	0.53	0.52	0.53
**TEES 2.1**	0.28	0.82	0.42
**IRISA**	0.36	0.46	0.40
**Boun**	0.21	0.38	0.27
**LIMSI**	0.04	0.19	0.06

The results obtained on the test set are reported.

Tables 7, 8, and 9 show the effects of the anaphora resolution module for localization extraction and the syntax rules for PartOf relation extraction on the training, development, and test sets, respectively. The first rows of these tables show the results obtained by the *Boun 2 *system. The second row shows the results obtained by removing the anaphora resolution module from the system, and the third and fourth rows show the results obtained by removing the first and second syntax rules from the system, respectively. The anaphora resolution module achieves a considerable increase in recall on all data sets (training, development, and test), which leads to improved F-score performances on the training and test sets. The two syntax rules have similar effects. In general, they lead to an increase in recall, which can improve F-score if the drop in precision is relatively less (e.g. on the training and test sets).

**Table 7:** Effects of Anaphora Resolution Module and Syntax Rules *(Localization and PartOf Event Extraction)*.

System	Recall	Precision	F-score
**Boun 2**	0.46	0.42	0.44
**- Anaphora**	0.36	0.45	0.40
**- Syntax rule 1**	0.45	0.42	0.43
**- Syntax rule 2**	0.46	0.42	0.44

The results obtained on the training set are reported.

**Table 8:** Effects of Anaphora Resolution Module and Syntax Rules *(Localization and PartOf Event Extraction)*.

System	Recall	Precision	F-score
**Boun 2**	0.55	0.40	0.46
**- Anaphora**	0.50	0.44	0.47
**- Syntax rule 1**	0.54	0.40	0.46
**- Syntax rule 2**	0.53	0.42	0.47

The results obtained on the development set are reported.

**Table 9:** Effects of Anaphora Resolution Module and Syntax Rules *(Localization and PartOf Event Extraction)*.

System	Recall	Precision	F-score
**Boun 2**	0.53	0.52	0.53
**- Anaphora**	0.46	0.56	0.50
**- Syntax rule 1**	0.52	0.52	0.52
**- Syntax rule 2**	0.50	0.55	0.52

The results obtained on the test set are reported.

These results demonstrate that performing a more fine-grained analysis of the text at the sentence level and incorporating an anaphora resolution module to handle relations that span multiple sentence is an effective approach for extracting relations in the bacteria biotopes domain. Our improved system achieves state-of-the-art results. However, there is still a lot of room for improvement. Our current approach assumes that if a specific bacteria (or its coreference) occur in the same sentence with a habitat entity, there is a localization relation between them. Deeper syntactic and semantic analysis of the sentences by using full or dependency parsing strategies can enhance the accuracy of the system. The PartOf relation extraction method that we proposed is only able to identify PartOf relations between habitat entities that overlap (e.g. *"human gastrointestinal tract" *and *"human"*). A deeper syntactic analysis can enable identifying long-distance relations between habitat entities (e.g. the PartOf relation between *"human" *and *"gut" *in the sentence *"This organism is found in humans as a normal component of gut flora."*). Furthermore, the lower accuracy of the PartOf relations may also be caused by the fact that our system does not take into account whether the candidate habitat entities are hosts or host parts. For example, the habitat entity *"fresh water" *is neither a host nor a host-part. Therefore, it should not be considered for a PartOf relation. Including a module that can pre-identify the habitats which can act as hosts or host-parts in advance, may improve the performance of the system for PartOf relation extraction.

## Conclusion

In this study, we present the systems that we developed for the Bacteria Biotope Task in the BioNLP Shared Task 2013, as well as the new methods that we developed and the improvements that we obtained after the official evaluation. We introduce a linguistically-motivated rule-based approach for Sub-task 1 that targets identifying and normalizing habitat entities through an ontology, and Sub-task 2 that targets extracting the localization and part-of events among the given bacteria and habitat entities. The Sub-task 1 and Sub-task 2 systems submitted to the shared task obtained promising results in the official evaluation. With the developments after the shared task, significant improvements are obtained in the performance of the Sub-task 2 system. The paragraph-based system submitted to the shared task is compared with the newly developed sentence-based system that includes an anaphora resolution module to handle relations with scope wider than a sentence. The new system achieves 53% F-score, which is not only significantly higher than the 27% F-score of the paragraph-based system, but also the best F-score performance obtained on the shared task test set so far. Several extensions are proposed for the Sub-task 1 system as well. Extending the candidate noun phrases by their modifiers resulted in lower performance, due to the prepositional phrase attachment ambiguity problem. Incorporating an ontology expansion module to our Sub-task 1 system did not lead to improvement in the performance in terms of SER score. The Boun and Boun 2 systems achieved the same SER value (68%), which is close to the SER value of the system that ranked first in the shared task.

This paper shows that our approaches based on the shallow syntactic analysis of the text and linguistically-motivated hand-coded rules are as effective as machine learning approaches for named entity detection, ontology-based normalization, and relation extraction in the bacteria biotopes domain. Our future directions for research include employing a full syntactic parsing approach to better identify the modifiers of the entities in Sub-task 1 and making use of dependency parsing in Sub-task 2 to handle long-distance PartOf relations, as well as to more accurately identify localization relations. We also plan to investigate the adaptation and evaluation of our proposed approaches for extracting bacteria biotope information from scientific publications.

## Competing interests

The authors declare that they have no competing interests.

## Authors' contributions

IK: Design and implementation of the algorithms, evaluation of the results, and drafting of the manuscript; AO: Design of the algorithms, evaluation of the results, and drafting of the manuscript. All authors read and approved the final manuscript.

## Declarations

Costs for publication were funded by Marie Curie FP7-Reintegration-Grants within the 7th European Community Framework Programme.

This article has been published as part of *BMC Bioinformatics *Volume 16 Supplement 10, 2015: BioNLP Shared Task 2013: Part 1. The full contents of the supplement are available online at http://www.biomedcentral.com/bmcbioinformatics/supplements/16/S10.
